# Stunting and Overweight among 12-24-Month-Old Children Receiving Vaccination in Ho Chi Minh City, Vietnam

**DOI:** 10.1155/2019/1547626

**Published:** 2019-02-19

**Authors:** Huynh Giao, Pham Le An, Nguyen Truong Vien, Tran Van Khanh, Bui Quang Vinh

**Affiliations:** ^1^Faculty of Public Health, University of Medicine and Pharmacy at Ho Chi Minh City, Vietnam; ^2^Center for training of Family Medicine University of Medicine and Pharmacy at Ho Chi Minh City, Vietnam; ^3^Dist 2 Hospital. Member of Vietnam Young Physician Association (VYPA), Vietnam; ^4^Department of Pediatrics, University of Medicine and Pharmacy at Ho Chi Minh City, Vietnam

## Abstract

**Introduction:**

Malnutrition and obesity are a double burden on children in developing countries and could induce higher risks of noncommunicable diseases in the long term. In the big cities of Vietnam, both issues are present and share the issue of nutrition problems; the prevalence of malnutrition in children is gradually decreasing while the prevalence of obesity is increasing rapidly. The paper aims to identify the prevalence of stunting and overweight/obesity in apparently healthy young children in Ho Chi Minh City (HCMC).

**Methods:**

A prospective cross-sectional study recruited 12-24-month-old children receiving national vaccination in community health centers in HCMC from February 2016 to July 2017. Sixteen healthcare centers were randomly selected among 8 districts of HCMC. Stunting and overweight were defined by height-for-age z-score <-2 SD and BMI z-score ≥+2 SD.

**Results:**

A total of 768 children had mean age of 16.8±4.2 months old, 51.7% boys. The prevalence of stunting and overweight/obesity was 8.2% and 10.7%, respectively. Stunting was associated with older age, boys, and low birth weight of children and occupation of mothers (P <0.05). No associated risk factor was observed for overweight/ obesity status.

**Conclusion:**

The prevalence of overweight/obesity was higher than the prevalence of stunting in 12-24-month-old children in HCMC. Overweight/obesity would be a public health problem for children in big cities.

## 1. Introduction

Stunting and obesity are a double burden for children in developing countries, especially in urban areas. Stunting in children may have significant disadvantages in the future such as learning difficulties, low income, and limited participation in the community and even affect the next generation. Overweight children may induce higher risks of noncommunicative diseases such as hypertension, lipid disorders, diabetes mellitus, and cardiovascular diseases in adults. In developing countries, while the prevalence of stunting was slowly declining in children, the prevalence of overweight and obese children is increasing rapidly. In Vietnam, both stunting and overweight children coexisted. The national prevalence of stunting (height-to-age z-score <-2 SD) in under-5-year-old children reduced from 43.3% in 2000 to 29.3% in 2010, but the prevalence of overweight (BMI z-score ≥+2 SD) increased from 2.6% in 2005 to 4.6% in 2010 [1, 2]. The increasing trends of overweight children and obesity were obvious in big cities. A longitudinal study in urban districts of Ho Chi Minh City showed that the prevalence of overweight children and obesity among adolescents was 12.4% and 1.7% in 2004 but increased to 16.7% and 5.1% in 2010, respectively [3].

The main purpose of this publication is to describe the prevalence of stunting and overweight in 12-to-24-month-old children who receive vaccination in the Expanded Program Immunization (EPI) in community health centers in Ho Chi Minh City (HCMC), Vietnam.

## 2. Population and Methods

### 2.1. Design

It was a prospective cross-sectional study in Ho Chi Minh City from February 2016 to July 2017.

### 2.2. Study Population

Eligible children were infants from 12 to 24 months receiving the EPI vaccination at community health centers in HCMC. Two health centers were randomly selected from each of eight districts, including 6 urban and 2 rural districts, in HCMC. A total of 768 infants were recruited from February 2016 to July 2017.

### 2.3. Data Collection

A structural questionnaire was developed and used to collect information. First, information on demographic characteristics of children and parents were collected by interviewing children's mothers or caregivers, using a structural and precoded questionnaire. Main sociodemographic variables included mother's age, address, occupation, education levels, income, numbers of children in household, and children's age, gender, birthweight, early breastfeeding, exclusive breastfeeding during the first 6 months of age, the age of diversification, and vaccination status.

Second, anthropometric measurements were obtained during the vaccination visit. Weight and length of children were measured by an infant stadiometer with the accuracy to 1 mm and a pediatric digital scale with the accuracy to 100 gram. Anthropometric data were standardized to z-scores of weight-for-age (WAZ), height-for-age (HAZ), weight-to-height (WHZ), and body mass index (BMIZ), using WHO Anthro software 3.2.2.

### 2.4. Variable Definitions

Stunting was defined as HAZ <-2 SD [4–6].

Overweight/obesity in children under 5 years of age was defined as BMIZ ≥ +2SD [4–6].

### 2.5. Statistical Method

Data was collated using Epidata 3.0, standardized using WHO Anthro 3.2.2, and analyzed using Stata 13 software. Continuous variables were estimated as mean (standard deviation), discrete variables as frequency and percentage. Comparisons of estimates between 2 groups were performed using the t-test for continuous variables and Chi-square or Fisher's exact test for discrete variables. Multivariate analysis for binary variables as stunting and overweight was performed using Poisson regression with selected variables with significant levels <0.20 in the binary analysis. Statistical P value was defined as <0.05.

### 2.6. Ethical Approval

Mothers or caregivers of eligible children agreed and gave informed consents before participation in the study. The study was approved by the Ethics Council of the University of Medicine and Pharmacy HCMC (protocol number 165/UMP-BOARD). Coding was applied to ensure the participant anonymity.

## 3. Results

### 3.1. Baseline Characteristics of Children and Mothers

A total of 768 children recruited in the study had mean age of 16.8 months, 51.7% were boys, 10.5% had low birth weight (Table 1). Their mothers were mainly in the age group from 25 to 40 years old (85%), public workers (55.1%), in high school level (55.0%), and living in urban areas (73.7%).

### 3.2. Anthropometric Measurements and Nutritional Status

Anthropometric indices were presented in Table 2. Participating children had a low prevalence of malnutrition with 2.5% underweight, 8.2% stunting, and 5.7% wasting status. The prevalence of overweight was higher at 10.7%.

### 3.3. Risk Factors of Stunting Status

Comparisons of characteristics of children and mothers in groups with and without stunting were presented in Table 3. The results of multivariate analysis were presented in and Table 4. Risk factors for stunting were higher age, boys, low birth weight, occupation, and urban address (P <0.05). Adjusting for other variables, boys had a 0.94 higher risk of stunting compared to girls; and low birth weight had 1.58 higher risk of stunting compared to normal birth weight. Mothers having occupations as retail workers (known as Sellers in Vietnam) and housewife had decreased risk of stunting children compared with mothers working in public sectors (OR 0.29 and 0.52, P<0.05, respectively).

### 3.4. Risk Factors of Overweight/Obesity Status

Comparisons of characteristics of children and mothers in groups with and without overweight/obesity were presented in Table 5. The overweight/obesity group had similar characteristics with a nonoverweight/obesity group (all P values >0.05).

## 4. Discussion

Our study showed that, in a population of 12-24-month-old apparently healthy children in Ho Chi Minh City, the prevalence of stunting was 8.2% and the prevalence of overweight/obesity was 10.7%.

Our result found the prevalence of stunting in small children in HCMC was lower than the prevalence of stunting in under-5-year children as 29.3% in a national survey reported in 2010 in Vietnam [1]. The rate of stunting in Ho Chi Minh City is lower than that whole country; this could be explained due to the younger age of our subjects, better nutritional conditions in HCMC as a big city, and many socioeconomic changes in the recent period. Previous studies have also shown that urban areas have a lower proportion of stunting than rural areas [7–10].

Our study showed that risk factors associated with stunting were older age, boy, low birth weight of children, and occupation of mothers. Stunting rates among boys were higher than those for girls. Similarly, stunting was associated with gender following studies of Gewa CA, Keino S, and Vonaesch [11–13]. Another study in healthy under-5-year children in Bangui and Madagascar in Africa showed similar to our research that stunting was associated with children's age [11]. In fact, many studies have also shown that the stunting rate tends to increase between 0 and 24 months [6, 14, 15]. This could be explained based on McCusker's research in Madagasca, the height-for-age in the group of 6-24-month-old children fluctuated more than the one in the overall 0-5-year-old children or in the older age group of 25-64-month-old children [16].

Low birth weight was an important risk factor related to children's nutritional status; it had a 1.58 higher risk of stunting compared to normal birthweight. This result was also consistent with other studies such as Cruz's research in Mozambique [17], Vitolo in Brazil [18], and Utami in Indonesia [19]. Therefore, it was necessary to care for maternal nutrition during pregnancy to reduce the risk of stunting. Mothers having occupations as sellers and housewives had a decreased risk of stunting children compared with mothers working in public sectors that could be that occupations were related to spending the time to care for and better care practice.

The prevalence of overweight children in our study in HCMC was higher than the prevalence of overweight in under-5-year children as 4.6% in the 2010 report in the National survey, Vietnam [1]. The result could be explained due to better nutritional status in children in HCMC as a big city and improving socioeconomic conditions in the recent period. Other studies have also shown that overweight/obesity rates in urban areas were higher than in rural areas; those living with better socioeconomic conditions had higher overweight and obesity rates [8, 20–22].

Our finding suggested a tendency of decreasing stunting and increasing overweight/obesity prevalence in big cities. In fact, overweight/obese rates would be a problem in the coming years in Vietnam; the characteristics of individuals, family, community, and society may mutually interact and contribute to the development of overweight/obesity in children [23]. In addition, game shops and fast food stores would be increasingly available in big cities while spaces for physical activity would be limited.

We did not find associated factors with the overweight status. In literature, overweight young children could be associated with age, sex, mother's educational level, family socioeconomic status, breastfeeding, and weight at birth [24–30]. The fact that no associated factor was observed in this study may be explained by the low sample size, young age at the vaccination, and poor accuracy of Vietnamese mothers in answering individual questions about socioeconomic status.

### 4.1. Limitations

Our study could have some limitations which affect the interpretations of our result. Our selection of 768 children from 8 districts in HCMC was random, but could not really be a representation for all children in the population in HCMC. We did not collect parental heights, which could be associated with stunting status of the children.

## 5. Conclusion

In 12-24-month children in HCMC in Vietnam, the prevalence of stunting was reduced to 8.2% but the prevalence of overweight/obesity was increased to 10.6%. Overweight/obesity would be a public health problem for children in big cities in Vietnam and needs specific measures for prevention.

## Figures and Tables

**Table 1:** Baseline sociodemographic and anthropometric characteristics of participating children and mothers (n=768).

Variables	Values*∗*
*Children*	
Age (months)	16.8 ± 4.2
Male	397 (51.7)
Low birth weight (< 2500 g)	81 (10.5)
Early breastfeeding (<1 hr after delivery)	482 (62.8)
Receiving complementary foods	
< 6 months	75 (9.8)
≥6 months	693 (90.2)

*Mothers*	
Age (years) *(n=767)*	
≥ 18 and <25	68 (8.9)
≥ 25 and <40	652 (85.0)
≥ 40	47 (6.1)
Occupation *(n=737)*	
Government officer/ worker	406 (55.1)
Seller	89 (12.1)
Housewife	242 (32.8)
Education *(n=767)*	
< Primary school	116 (15.1)
Secondary school	229 (29.9)
> High school	422 (55.0)
Gross household income *(n=727)*	
Moderate	691 (95.1)
Poor, near-poor households	36 (4.9)
Number of children in household *(n=767)*	
1	375 (48.9)
2	318 (41.5)
≥ 3	74 (9.6)
Residence location* (n=767)*	
Rural	202 (26.3)
Urban	565 (73.7)

*∗*Values are mean ± SD or frequency (percent).

**Table 2:** Anthropometric measurements and nutritional status of children*∗* (n=768).

Variables	Values
Weight-for-age Z-score (WAZ)	0.28 ± 1.16
Length/height-for-age Z-score (HAZ)	0.06 ± 1.52
Weight-for-length/height Z-score (WHZ)	0.19 ± 1.38
Body Mass Index Z-score (BMIZ)	0.34 ± 1.43
Underweight (WA< -2SD)	19 (2.5)
Stunting (HA< -2SD)	63 (8.2)
Wasting (WH< -2SD)	44 (5.7)
Overweight and Obesity (BMI ≥ +2SD)	82 (10.7)
Overweight (+2SD ≤ BMI < +3SD)	61 (8.0)
Obesity (BMI ≥ +3SD)	21 (2.7)

*∗*Values are mean ± SD or frequency (percent).

**Table 3:** Characteristics of children and mothers in children groups with stunting and without stunting*∗* (n=768).

	Stunting	No-stunting	P-value
	(N=63)	(N=705)	
*Children*			
Gender			<0.05
Female	20 (31.8)	351 (49.8)	
Male	43 (68.2)	354 (50.2)	
Age (months)	18.7 ± 4.3	16.6 ± 4.2	<0.001*∗∗∗*
Weight at birth			<0.001
Low (< 2500 g)	16 (25.4)	65 (9.2)	
Normal (≥ 2500 g)	47 (74.6)	640 (90.8)	
Breastfeeding in the first hour after birth	45 (71.4)	437 (62.1)	0.14
Yes	45 (71.4)	437 (62.1)	
No	18 (28.6)	267 (37.9)	
Receiving conmplementary foods			
< 6 months	5 (7.9)	70 (9.9)	0.61
≥6 months	58 (92.1)	635 (90.1)	

*Mothers*			
Age (years) *(n=767)*			0.85*∗∗*
≥ 18 and <25	4 (7.9)	63 (8.9)	
≥ 25 and <40	55 (87.3)	597 (84.8)	
≥ 40	3 (4.8)	44 (6.3)	
Occupation *(n=737)*			0.02
Government officer/ worker	43 (71.7)	363 (53.6)	
Seller	3 (5.0)	86 (12.7)	
Housewife	14 (23.3)	228 (33.6)	
Education *(n=767)*			0.21
< Primary school	6 (9.5)	110 (15.6)	
Secondary school	16 (25.4)	213 (30.3)	
> High school	41 (65.1)	381 (54.1)	
Gross household income *(n=727)*			0.63*∗∗*
Moderate	59 (95.2)	632 (95.0)	
Poor, near-poor households	3 (4.8)	33 (5.0)	
Number of children in household *(n=767)*			0.69
1	33 (52.4)	342 (48.6)	
2	23 (36.5)	295 (41.9)	
≥ 3	7 (11.1)	67 (9.5)	
Residence location* (n=767)*			<0.05
Rural	10 (15.9)	192 (27.3)	
Urban	53 (84.1)	512 (72.7)	

*∗* Values are mean ± SD or frequency (percent) and P values of Chi-square test, *∗∗* Fisher exact, and *∗∗∗* t-tests used to compare groups with and without stunting, excluding missing data.

**Table 4:** Risk factors associated with stunting status in multivariate analysis.

Variables	Risk Ratio	95% CI	p-value
Gender of infants (male)	1.94	1.17-3.23	*<0.05*
Age	1.08	1.03-0.15	*<0.05*
Low birth weight	2.58	1.54-4.34	*<0.001*
Breastfeeding in the first hour after birth	1.58	0.92-2.71	0.09
Occupation			
Worker	1		
Seller	0.29	0.10-0.90	*<0.05*
Housewife	0.52	0.29-0.95	*<0.05*
Residence location (urban)	1.45	0.72-2.94	0.30

**Table 5:** Characteristics of children and mothers in children groups with overweight/obesity and without overweight/obesity (n=768).

	Overweight/obesity		p value*∗*
	Yes (N= 82)	No (N= 686)	
*Characteristics of children*			
Gender			
Female	40 (48.8)	331 (48.2)	0.93
Male	42 (51.2)	355 (51.8)	
Age (months)	17.4 ± 4.3	16.5 ± 4.2	0.19
Weight at birth			
Low (< 2500 g)	9 (11.0)	72 (10.5)	0.89
Normal (≥ 2500 g)	73 (89.0)	614 (89.5)	
Breastfeeding in the first hour after birth			
Yes	51 (62.2)	431 (62.9)	0.90
No	31 (38.8)	254 (37.1)	
Receiving conmplementary foods *(n=219)*			
< 6 months	10 (12.2)	65 (9.5)	0.43
≥ 6 months	72 (87.8)	621 (90.5)	

*Characteristics of mothers*			
Age (years) *(n=767)*			
≥ 18 and <25	3 (3.7)	65 (9.5)	0.17
≥ 25 and <40	75 (91.5)	577 (84.2)	
≥ 40	4 (4.8)	43 (6.3)	
Occupation *(n=737)*			
Government officer/ worker	48 (59.3)	358 (54.6)	0.38
Seller	6 (7.4)	83 (12.7)	
Housewife	27 (33.3)	215 (32.7)	
Education *(n=767)*			
< Primary school	13 (15.9)	103 (15.0)	0.60
Secondary school	28 (34.1)	201 (29.3)	
> High school	41 (50.0)	381 (55.6)	
Gross household income *(n=727)*			
Moderate	74 (91.4)	617 (95.5)	0.10
Poor, near-poor households	7 (8.6)	29 (4.5)	
Number of children in household *(n=767)*			
1	34 (41.5)	341 (49.8)	0.16
2	42 (51.2)	276 (40.3)	
≥ 3	6 (7.3)	68 (9.9)	
Residence location* (n=767)*			
Rural	26 (31.7)	176 (25.7)	0.24
Urban	56 (68.3)	509 (74.3)	

*∗* Chi–square, *∗∗* t- tests used to comparison between overweight/obesity and without overweight/obesity groups.

## Data Availability

The primary data used to support the findings of this study are available from the corresponding author upon request.

## Supplementary Materials

Supplementary Materials Dataset used for our study was attached. Variables were listed corresponding to the variables name within () in STATA data file. Children's variables including age (tuoi_thang), sex (q2moi), birth weight (q6moi), early breastfeeding (q9moi), receiving complementary foods (bot), weight-for-age Z-score (zwa), length/height-for-age Z-score (zha), weight-for-length/height Z-score (zwh), body mass index Z-score (zbmi), underweight (nhecan), stunting (thapcoi), wasting (teocom), and overweight and obesity (thuacanmoibeophi). Mother's variables including age (q15moi), occupation (q19moi), education (q20moi), gross household income (thunhap_gr), number of children in household (q22moi), and residence location (q18).
